# Acknowledgement to Reviewers of *Animals* in 2017

**DOI:** 10.3390/ani8010009

**Published:** 2018-01-09

**Authors:** Animals Editorial Office

**Affiliations:** MDPI AG, St. Alban-Anlage 66, 4052 Basel, Switzerland

Peer review is an essential part in the publication process, ensuring that *Animals* maintains high quality standards for its published papers. In 2017, a total of 100 papers were published in the journal. Thanks to the cooperation of our reviewers, the median time to first decision was 23 days and the median time to publication was 64 days. The editors would like to express their sincere gratitude to the following reviewers for their time and dedication in 2017:
Adamczyk, KrzysztofKuhar, ChrisAllen, KateKurt, TimothyAllen, MichaelLassen, JesperAmpe, BartLaurence, MichaelAnagnostopoulos, GeorgiosLaven, RichardAndersen, MonicaLeach, MatthewAnderson, KennethLewis, DaveAparicio, Miguel A.Lopez-Salesansky, NoeliaArcher, Gregory S.Lovercamp, KyleArhant, ChristineMacKay, JillArkins, SeanMacleod, Malcolm R.Arney, DavidMahony, Timothy JohnAtes, SerkanMain, DavidAzarpajouh, SamanehMalashichev, YegorBackus, Brittany L.Maltz, EphraimBacon, HeatherManning, LouiseBaker, SandraManteca, XavierBaker, KathyMaple, TerryBarbieri, SaraMargerison, JeanBarnard, ShanisMarinelli, LietaBarnett, MarkMarsa-Sambola, FerranBattini, MonicaMartelli, GiovannaBauer, ErikaMartin, JessicaBaumans, VeraMatchock, Robert L.Beatty, JuliaMather, JenniferBeck, AlanMatsuoka, AtsukoBennett, Elizabeth L.McGlone, JohnBentosela, MarianaMcLennan, KristaBerezowski, JohnMcMillin, Kenneth W.Berliner, ElizabethMeagher, RebeccaBiagi, Pier FrancescoMeehan, Cheryl L.Binanti, DianaMeijboom, FranckBjalobok, FaithMellor, DavidBland, IanMench, JoyBoe-Hansen, GryMergenthaler, MarcusBolea, RosaMeyer, IbenBolwell, CharlotteMeyer, Robert E.Boogaard, BirgitMichalopoulou, EleniBoone, JohnMichie, CraigBoyle, LauraMiele, MaraBrady, Colleen M.Molento, Carla Forte MaiolinoBrearley, JackieMontoya Alonso, Jose AlbertoBrook, RyanMooring, MikeBroom, DonaldMorgan, KathleenBuning, J.T. De CockMorgan-Davies, ClareBurghardt, GordonMorris, Kevin N.Bushby, Philip A.Morton, DavidButterworth, AndrewMuboko, NeverBüttner, KathrinMurison, PamelaCalambokidis, JohnNagy, AnnamariaCannon, ClaireNannoni, EleonoraCarney, Hazel C.Napolitano, FabioCarter, AnneNg, ZenithsonCenci-Goga, Beniamino T.Noonan, MichaelChandler, Cynthia KayOppedal, FrodeChastant-Maillard, SylvieOsinga, Sjoukje A.Cheng, Heng-WeiO'Sullivan, SiobhanChur-Hansen, AnnaPanagiotopoulou, OlgaCirulli, FrancescaParanhos Da Costa, Mateus José RodriguesCitek, JindrichParkin, TimClarke, NancyPasicka, EdytaCole, MatthewPassantino, AnnamariaCoombe, JoPatronek, GaryCorazzin, MircoPatterson, PaulCosta, MarcioPearce, ChristopherCostall, AlanPemberton, NeilD’Occhio, MichaelPeterson, SamDai, FrancescaPhalen, DavidDalla Villa, Paolo FelicePiccolo, JohnDallaire, Jamie AhloyPirrone, FedericaD’Aniello, BiagioPlà, Lluis M.Dashper, KatePound, PandoraDavies, AnnaProgar, Amber AdamsDe Rensis, FabioProtopopova, AlexandraDelgado, MikelProudfoot, KathrynDemyda-Peyrás, SebastiánQuattrocchi, FedoraDeVries, TrevorRast, LuziaDi Giminiani, PierpaoloRawlings, John MerrittDixon, LauraReese, LauraDoneley, BobReimert, InongeDonnett, UriRidoutt, BradDórea, FernandaRiley, ChristopherDoughty, AmandaRogers, Chris W.Doveren, AldoRogers, LesleyDowsey, AndrewRollin, BernardDunlop, RebeccaRowan, AndrewDvir, HayRuch Gallie, RebeccaDwyer, CathyRuhnke, IsabelleDzidic, AlenSaleri, RobertaEdinboro, Charlotte H.Sant'Ana, Manuel MagalhãesErasmus, Marisa A.Scheerlinck, Jean-PierreErrington, Timothy M.Schinckel, Allan PFàbrega, EmmaSchmoelzl, SabineFarnworth, Mark J.Schoster, AngelikaFawcett, AnneSchwarzer, AngelaFei, Andrew Chang-YoungScobie, DavidFerrante, ValentinaScollo, AnnalisaFidani, CristianoScullion Hall, LauraFine, AubreyShaw, DavidFishlock, VickiShivley, Chelsey B.Font I Furnols, MariaShriver, AdamFranco, Nuno HenriqueSiegford, JaniceFreeman, LynettaSlater, Margaret R.Freire, RafSmak, J.A.Galindo, FranciscoSneddon, JenniferGallis, ChristosSparrey, JulianGamborg, ChristianStaaveren, Nienke VanGardiner, AndrewStadig, Lisanne M.Garry, FranklynStafleu, FransGatel, LaureStatham, PoppyGebremedhin, KifleSteibel, Juan PedroGeers, RonyStella, JudiGeorg, HeikoStephenson, Mitchell B.Gianguzza, PaolaStoeger, AngelaGiersberg, Mona FranziskaSturaro, E.Gill, MargaretSwinker, Ann M.Gillett, JamesSzabo, JuditGingerich, EricTadich, TamaraGingrich, Elise N.Tan, BieGlenk, Lisa MariaTeague, RichardGold, JeniferThornber, PeterGrajfoner, DashaThornton-Kurth, KaraGrandin, TempleTilbrook, AlanGrimm, HerwigTiplady, CatherineGrogono-Thomas, RosemaryTodd, TeriGuzek, DominikaToribio, Jenny-AnnHa, James CTribe, AndrewHadley, PhilTu, YanHagen, JennyTucker, CorrinaHall, SophieTurner, MarkHall, CarolUwiera, RichardHanlon, AlisonVan Eerdenburg, Frank J C MHargis, BillyVan Heezik, YolandaHart, BenjaminVentura, BethHarvey, NaomiVerdon, MeganHässig, MichaelVerstegen, Martin W. A.Hassink, JanVevea, JackHaxel, JoeVon Essen, EricaHazel, SusanVoslarova, EvaHeleski, CamieWaldmann, Karl HeinzHenry, BeverleyWalk, Carrie L.Hernandez, Carlos E.Wallis, LisaHerskin, Mette S.Wang, K.Hiby, EllyWatson, Andrea K.Hickson, RebeccaWebster, JohnHoffman, ChristyWellbrock, WiebkeHokkanen, Ann-HelenaWels, H.Houpt, Katherine AlbroWeng, Hsin-YiHoward, VeronicaWerner, Scott J.Howell, TiffaniWhite, GavinHumer, ElkeWhite, StevenHunt, MelissaWhite, RachelIjichi, CarrieWhite, PeterIrvine, LeslieWhiting, TerryIzmirli, SerdarWhitley, David S.Jacobs, LeonieWhittaker, AlexandraJohnson, Jay S.Widmar, Nicole OlynkJonker, ArjanWilliams, Scott C.Karsten, CynthiaWilliams, AngelicaKazdin, AlanWilson, DavidKeates, HelenWindschnurer, InesKiddie, JennaWinkel, AlbertKjaer, JoergenWohlfarth, RainerKnight, AndrewWycislo, KathrynKnight, PeterWynne, CliveKoluman-Darcan, NazanYamauchi, HiroyukiKongara, KavithaYeates, JamesKortet, RaineZaefarian, FifiKrawczel, Peter D.Zervas, GeorgeKreisler, RachaelZivotofsky, Ari

